# Suspected recurrence of brain metastases after focused high dose radiotherapy: can [^18^F]FET- PET overcome diagnostic uncertainties?

**DOI:** 10.1186/s13014-016-0713-8

**Published:** 2016-10-21

**Authors:** Alexander Romagna, Marcus Unterrainer, Christine Schmid-Tannwald, Matthias Brendel, Jörg-Christian Tonn, Silke Birgit Nachbichler, Alexander Muacevic, Peter Bartenstein, Friedrich-Wilhelm Kreth, Nathalie Lisa Albert

**Affiliations:** 1 Department of Neurosurgery, Ludwig-Maximilians-University of Munich, Munich, Germany; 2 Department of Nuclear Medicine, Ludwig-Maximilians-University of Munich, Marchioninistr. 15, 81377 Munich, Germany; 3 Institute of Clinical Radiology, Ludwig-Maximilians-University of Munich, Munich, Germany; 4 Department of Radiation Oncology, Hospital of the University of Munich, Campus Grosshadern, Ludwig-Maximilians-University of Munich, Munich, Germany; 5 European CyberKnife Center Munich, Munich, Germany

**Keywords:** Brain metastases, Radiosurgery, ^18^F-FET PET, Kinetic analysis, Radionecrosis

## Abstract

**Background:**

After focused high dose radiotherapy of brain metastases, differentiation between tumor recurrence and radiation-induced lesions by conventional MRI is challenging. This study investigates the usefulness of dynamic *O*-(2-^18^F-Fluoroethyl)-L-Tyrosine positron emission tomography (^18^F-FET PET) in patients with MRI-based suspicion of tumor recurrence after focused high dose radiotherapy of brain metastases.

**Methods:**

Twenty-two patients with 34 brain metastases (median age 61.9 years) were included. Due to follow-up scan evaluations after repeated treatment in a subset of patients, a total of 50 lesions with MRI-based suspicion of tumor recurrence after focused high dose radiotherapy could be evaluated. ^18^F-FET PET analysis included the assessment of maximum and mean tumor-to-background ratio (TBR_max_ and TBR_mean_) and analysis of time-activity-curves (TAC; increasing vs. decreasing) including minimal time-to-peak (TTP_min_). PET parameters were correlated with histological findings and radiological-clinical follow-up evaluation.

**Results:**

Tumor recurrence was found in 21/50 cases (15/21 verified by histology, 6/21 by radiological-clinical follow-up) and radiation-induced changes in 29/50 cases (5/29 verified by histology, 24/29 by radiological-clinical follow-up). Median clinical-radiological follow-up was 28.3 months (range 4.2–99.1 months). ^18^F-FET uptake was higher in tumor recurrence compared to radiation-induced changes (TBR_max_ 2.9 vs. 2.0, *p* < 0.001; TBR_mean_ 2.2 vs. 1.7, *p* < 0.001). Receiver-operating-characteristic (ROC) curve analysis revealed optimal cut-off values of 2.15 for TBR_max_ and 1.95 for TBR_mean_ (sensitivity 86 %, specificity 79 %). Increasing TACs and long TTP_min_ were associated with radiation-induced changes, decreasing TACs with tumor recurrence (*p* = 0.01). By combination of TBR and TACs, sensitivity and specificity could be increased to 93 and 84 %.

**Conclusions:**

In patients with MRI-suspected tumor recurrence after focused high dose radiotherapy, ^18^F-FET PET has a high sensitivity and specificity for the differentiation of vital tumor tissue and radiation-induced lesions.

## Background

Focused high dose radiotherapy, such as stereotactic radiosurgery (SRS), is increasingly used for small sized brain metastases especially in the “oligometastases situation” [1, 2]. Low-activity iodine-125 brachytherapy (SBT) is another ablative strategy which has been shown to be effective especially in recurrent brain metastases after previously performed radiotherapy [3].

The treatment rationale of both SRS and SBT is to obtain tumor control through early cytocidal effects as well as late vascular changes [4]. However, lesions that undergo such treatment constitute a diagnostic challenge: in conventional follow-up magnetic resonance imaging (MRI) a new ring-shaped contrast enhancement can arise at the site of the highest delivered dose as indication of blood-brain barrier (BBB) disruption. These lesions will either expand or resolve over time [5–7]. Therefore, conventional MRI cannot adequately distinguish between tumor recurrence and treatment response [8]. Recent data suggest that molecular imaging techniques might help to overcome such limitations in intracranial metastases which had previously undergone focused high dose radiotherapy. Whereas positron emission tomography (PET) with the widely used ^18^F-2-deoxy-2-fluoro-D-glucose (^18^F-FDG) has low diagnostic accuracy after SRS, the use of radiolabelled amino acids or amino acid analogues such as L-methyl-^11^C-methionine (^11^C-MET) and O-(2-^18^F-Fluoroethyl)-L-Tyrosine (^18^F-FET) reaches sensitivity and specificity values in the range of 78 and 100 % rendering especially ^18^F-FET a highly reliable tracer in glioma imaging [9–14].

Still, there is only limited data on the diagnostic impact of ^18^F-FET PET in intracranial metastases, especially in case of previous exclusive focused high dose radiotherapy [12, 15]. For further clarification, we objected to evaluate if ^18^F-FET PET can differentiate adequately between vital tumor recurrence and radiation-induced lesions.

## Materials and methods

### Patients

Twenty-two patients (median age 61.9 years; 11female/11 male) with a suspicion of a recurrence of their brain metastases after previously performed focused high dose radiotherapy (i.e. SRS and SBT) were included. To rule out potentially confounding effects of WBRT in addition to focused high dose radiotherapy on ^18^F-FET uptake, we excluded all patients with a history of whole brain radiotherapy. Thirteen patients suffered from of a single tumor and 9 patients from multiple metastases (3 lesions in 3 patients, 2 lesions in 6 patients), which resulted in 34 evaluable lesions.

Static and dynamic ^18^F-FET PET was performed in all these patients at the time of the suspected tumor recurrence. The results of ^18^F-FET PET investigation and their classification as either concordant or discordant to the actual tumor status referred either to the corresponding tissue diagnosis obtained from PET-guided biopsy procedures at the time of suspected recurrence and/or to the results of clinical/radiological follow up evaluation. In patients with deteriorating neurological status and/or further lesion growth with steroid resistant edema after high dose radiotherapy histological evaluation was aimed whenever possible. Lesions exhibiting unchanged or regressive MRI findings and a stable/improved clinical performance over a time period of ≥ 6 months after the date of the suspected tumor recurrence were classified as stable disease. When there were new suspicious imaging changes of a lesion and/or clinical deterioration after a ≥ 6 months time interval of stable/regressive disease after the initial treatment, this suspicion was handled as new case with subsequent new ^18^F-FET PET investigation and new follow-up. Each individual metastasis per patient was evaluated. Criteria for recurrence were based on the Macdonald criteria [16]: i) the appearance of a new contrast enhancing area after previously observed complete response or ii) an increase of the enhancing area (>25 %) after previously observed partial response/stable disease. Accordingly, an overall number of 16 metastases (8 patients) were repeatedly analysed at different stages of the brain disease. Seven out of these 16 tumors underwent re-treatment with focused high dose radiotherapy (time interval between first and second treatment: 25 months). In the remaining nine lesions, ^18^F-FET PET re-evaluation was initiated due to an increase in contrast enhancement and/or steroid-resistant symptomatic edema after a median latency period of 8.6 months. Of these 9 lesions, 6 were found to be tumor recurrences and 3 were found to be radiation induced lesions (median follow up 23.1 months; range 13.6–33.9 months). Thus, a total number of 50 brain lesions was analysed.

Clinical parameters were determined using patients’ electronic medical records and paper charts. Patients were evaluated and consented by both an experienced neurooncologist (FWK) and nuclear medicine physician (NLA). The study was approved by the institutional review board. All patients had given written informed consent.

### Radiosurgery, iodine-125 stereotactic brachytherapy and stereotactic biopsy

Three-dimensional planning for SRS and SBT was routinely based on computerized tomography (CT) and MRI (slice thickness <3 mm). The treatment protocol for SRS was used as described in Schüttrumpf et al. [2]. In brief, the clinical target volume was set equivalent to the gross tumor volume. Expansion of the gross tumor volume with 1–2 mm resulted in the planning target volume. Radiation dose ranged from 18 to 24 Gy. For SBT, the definition of the treatment volume and the treatment planning was done as described in Schwartz et al. using the BrainLab AG Target software (version 1.19) [17]. The prescribed reference dose calculated to the outer tumor rim was generally 54.0 Gy. The dose rate was < 15.0 cGy/h. Prior to SBT, the diagnosis of a metastasis was confirmed with stereotactic biopsy as previously described [18]. Whenever the attending neuropathologist could make the diagnosis of a vital metastasis using smear specimen intraoperatively, SBT was carried out immediately. In case of intraoperative uncertainty regarding the correct diagnosis, SBT was withheld and the results of the paraffin embedded analysis was awaited (all sections were stained with hematoxylin and eosin). Another CT scan was done at the first postoperative day to demonstrate the correct position of the implanted seed(s).

### ^18^F-FET PET image acquisition and evaluation

Dynamic ^18^F-FET PET image acquisition was performed over 40 min after injection of 180 MBq ^18^F-FET as described previously at a Siemens ECAT EXACT HR+ PET scanner [6, 19]. Image data were transferred to a HERMES workstation. ^18^F-FET PET analysis was performed for each lesion and included the assessment of the maximum standardized uptake value (SUV) as tumor-to-background ratio (TBR_max_), the mean tumor-to-background ratio (TBR_mean_) and dynamic analysis of tumoral ^18^F-FET uptake over time (increasing vs. decreasing time-activity-curves (TACs)). For the determination of background activity, a region-of-interest was drawn in six subsequent slices in the contralateral hemisphere including grey and white matter, added to a volume-of-interest, and the mean SUV was set as background acitivity. The TBR_max_ and TBR_mean_ were calculated by using the maximum and mean SUV within a semiautomatically drawn, threshold-based volume-of-interest (1.8 x background activity). For the dynamic analysis, a 90 % isocontour threshold region-of-interest was drawn in the 10–30 min summation images on each slice of suspicious uptake, and the respective TACs for the whole dataset were extracted. TACs within a 40 min time interval after tracer injection were defined as follows: (i) lesions with homogeneously increasing TACs with SUV constantly ascending or reaching a peak followed by a plateau in the subsequent frames was classified as having increasing TACs and (ii) lesions with homogeneously or heterogeneously decreasing TACs with SUV showing an early peak within the first 20 min time interval followed by a constant descent thereafter were classified as having decreasing TACs. Early fluctuations in the TACs within the first short time frames (7 × 10 s followed by 3 × 30 s) representing noise were excluded from kinetic analyses. For each slice within the tumor, the frame with the peak uptake was identified. The starting time of the frame plus half the frame duration, corresponding to the respective peak value, was set as time to peak (TTP). The shortest TTP present in at least 2 adjacent slices was defined as minimal TTP (TTP_min_) as described in Jansen et al. [20].

PET findings were evaluated by consensus reading of two experienced nuclear medicine physicians (NLA and MU) who were blinded for clinical and histological data as previously described [21].

### MR imaging

Clinical evaluations were combined with MRI at regular 3-month intervals. MRI protocols consisted of T1-weighted ± gadolinium contrast medium, T2-weighted and fluid attenuated inversion recovery (FLAIR) sequences. Slice thickness was 1.0 mm for all MRI sequences and all sequences were reconstructed in axial, coronal and sagittal planes.

### Statistical analysis

SPSS for Windows (SPSS, Version 21.0, Chicago, IL) was used for statistical calculations. The evaluated PET parameters (TBR_max_ and TBR_mean_ and increasing vs. decreasing time activity curves) were correlated with histopathological results when available and with the clinical follow-up. The comparison between tumor recurrence and radiation-induced lesion was performed using the Mann-Whitney-U test for continuously scaled variables and using the χ^2^ test for categorical variables. Continuous parameters were reported as mean ± standard deviation and range. A two-tailed *p*-value < 0.05 was considered significant. Receiver operating characteristic (ROC) analyses were performed in order to identify the optimal TBR_max_ cut-off value for the discrimination between tumor recurrence and radiation-induced lesion by choosing the cut-off leading to the highest product of sensitivity × specificity. Length of local PFS was calculated from the date of radiosurgery and analysed with the Kaplan-Meier method.

## Results

Patient characteristics and ^18^F-FET PET results are summarized in Table 1. Tumor recurrence was found in 21/50 cases (15/21 verified by histology, 6/21 by radiological-clinical follow-up) and radiation-induced lesions in 29/50 cases (5/29 verified by histology, 24/29 by radiological-clinical follow up data are included in Table 1). Median clinical and radiological follow up was 28.3 months (range 4.2–99.1 months; individual follow up data are indicated in Table 1), one patient was lost to follow-up. Median time from focused high dose radiotherapy to ^18^F-FET PET was 13.7 months (range 1.9–90.3 months). Within the first year, 18/29 (62.1 %) radiation-induced lesions and 10/21 (47.6 %) tumor relapses occurred. After two years, 27/29 (93.1 %) radiation-induced lesions and 20/21 (95.2 %) tumor relapses were seen. The rate of tumor relapse and tumor recurrence within the first 12 or 24 months did not differ (*p* = 0.23 and *p* = 0.62).

**Table 1:** Patient characteristics

Lesion number	Location	Histology	Previous RT	TBR max	TBR mean	TAC	TTP	Months between RT and PET	Final diagnosis	Diagnostic determinant	Follow up (months)
1	R parietal	NSCLC	SRS	1.5	1.4	n.a.	n.a.	1.9	Radiation induced lesion	Follow up	9.4
2	L insular	Malignant melanoma	SBT	3.4	2.3	Decreasing	12.5	2.1	Tumor	Follow up	14.8
3	L insular	Malignant melanoma	SRS	3.4	2.3	Increasing	35	2.2	Tumor	Histology	4.2
4	L parietooccipital	NSCLC	SBT	2.1	1.9	Decreasing	25	2.4	Tumor	Histology	29.9
5	L occipital	NSCLC	SBT	2.8	2.1	Increasing	35	2.5	Radiation induced lesion	Follow up	6.7
6	L frontal	Malignant melanoma	SBT	4.1	2.5	Increasing	35	2.6	Tumor	Follow up	4.6
7	L parietal	CUP	SRS	1.8	1.8	Increasing	35	3	Radiation induced lesion	Follow up	4.9
8	L pontine	Breast cancer	SRS	1.5	1.3	n.a.	n.a.	4.6	Radiation induced lesion	Follow up	30.9
9	R temporal	Malignant melanoma	SRS	2.1	1.9	Increasing	17.5	4.7	Radiation induced lesion	Follow up	16
10	L frontal	Malignant melanoma	SRS	2.0	1.9	Decreasing	17.5	4.7	Tumor	Histology	16
11	L cerebellar	Breast cancer	SRS	1.7	1.2	Increasing	35	4.7	Radiation induced lesion	Follow up	30.9
12	R parietal	Malignant melanoma	SRS	1.4	1.0	n.a.	n.a.	4.8	Radiation induced lesion	Follow up	6.8
13	R parietal	NSCLC	SRS	1.5	1.4	n.a.	n.a.	5.3	Tumor	Histology	9.4
14	L insular	Malignant melanoma	SBT	3.2	2.3	Decreasing	17.5	5.4	Radiation induced lesion	Follow up	14.8
15	R temporal	NSCLC	SRS	1.5	1.3	n.a.	n.a.	6	Radiation induced lesion	Follow up	13.6
16	L occipital	NSCLC	SRS	2.8	2.1	Decreasing	12.5	6	Tumor	Histology	13.6
17	L temporal	Malignant melanoma	SRS	2.9	2.1	Increasing	35	6	Radiation induced lesion	Follow up	33.8
18**	R frontal	Malignant melanoma	SRS	1.4	1.3	Increasing	35	8	Radiation induced lesion	Follow up	42.5
19	R cerebellar	Gastrointestinal cancer	SRS	2.3	2.0	Decreasing	12.5	8.5	Tumor	Histology	18
20	L frontal	NSCLC	SRS	1.9	1.8	Increasing	35	8.6	Radiation induced lesion	Histology	35.1
21	R frontal	NSCLC	SRS	1.6	1.4	Increasing	35	8.7	Radiation induced lesion	Histology	43.1
22	L frontal	NSCLC	SRS	1.2	1.0	n.a.	n.a.	8.7	Radiation induced lesion	Follow up	43.1
23	L frontal	Malignant melanoma	SRS	2.4	2.0	Decreasing	25	9.1	Tumor	Histology	22.2
24	R frontal	Gastrointestinal cancer	SRS	2.0	1.9	Increasing	25	9.3	Radiation induced lesion	Histology	17.1
25	R frontal	NSCLC	SRS	2.0	1.9	Decreasing	25	9.3	Radiation induced lesion	Histology	23.8
26	R temporal	NSCLC	SRS	1.5	1.1	n.a.	n.a.	9.4	Radiation induced lesion	Follow up	13.6
27	L insular	Malignant melanoma	SBT	3.5	2.4	Decreasing	7.5	10.5	Radiation induced lesion	Follow up	14.8
28	L parietal	Malignant melanoma	SBT	2.5	2.1	Decreasing	12.5	12.3	Tumor	Histology	14.5
29	R temporal	Malignant melanoma	SRS	1.5	1.2	n.a.	n.a.	14	Radiation induced lesion	Follow up	16
30*	L frontal	Malignant melanoma	SRS	3.5	2.3	Decreasing	7.5	14	Tumor	Histology	16
31	L insular	Malignant melanoma	SBT	3.5	2.4	Decreasing	12.5	14.7	Radiation induced lesion	Follow up	14.8
32	R occipital	Breast cancer	SRS	1.8	1.8	Increasing	35	15	Radiation induced lesion	Follow up	79.2
33	R frontal	Breast cancer	SRS	2.0	1.9	Increasing	35	15	Radiation induced lesion	Follow up	79.2
34	L cerebellar	Breast cancer	SRS	1.8	1.7	Increasing	35	15	Radiation induced lesion	Follow up	79.2
35	L occipital	NSCLC	SRS	3.0	2.1	Decreasing	4	15.8	Tumor	Histology	25.1
36	L frontal	Malignant melanoma	SRS	2.1	1.9	Increasing	35	18.2	Radiation induced lesion	Follow up	24.1
37	R frontal	NSCLC	SRS	3.0	2.2	Decreasing	12.5	18.6	Tumor	Follow up	23.8
38	R frontal	Malignant melanoma	SRS	3.5	2.2	Increasing	25	19.8	Tumor	Follow up	42.5
39	R frontal	Malignant melanoma	SRS	2.2	2.0	Decreasing	4	19.8	Tumor	Follow up	42.5
40	L cerebellar	Breast cancer	SRS	3.8	2.7	Increasing	35	19.9	Tumor	Histology	30.9
41	L pontine	Breast cancer	SRS	4.3	2.8	Decreasing	25	19.9	Tumor	Histology	30,9
42	L frontal	Malignant melanoma	SRS	2.0	1.9	Decreasing	17.5	20.3	Radiation induced lesion	Follow up	24.1
43	L frontal	Malignant melanoma	SRS	2.4	2.0	Decreasing	25	20.8	Tumor	Histology	22.2
44	R frontal	NSCLC	SRS	3.9	2.6	Decreasing	12.5	22.6	Tumor	Follow up	23.8
45	R frontal	Breast cancer	SRS	1.5	1.4	Decreasing	25	22.9	Radiation induced lesion	Follow up	33
46	L cerebellar	Breast cancer	SRS	2.4	2.0	Decreasing	17.5	22.9	Tumor	Histology	33
47	R cerebellar	NSCLC	SRS	2.6	2.0	Decreasing	17.5	23.5	Radiation induced lesion	Histology	33.6
48	R frontal	Malignant melanoma	SRS	1.4	1.3	Decreasing	4	28.4	Radiation induced lesion	Follow up	42.5
49	L parietal	NSCLC	SRS	1.7	1.5	Increasing	35	42.2	Radiation induced lesion	Follow up	51.5
50	R cerebellar	Renal cell carcinoma	SRS	2.9	2.1	Decreasing	17.5	90.3	Tumor	Histology	99.1

(*Fig. 1)

(**Fig. 2)

### Comparison of SUV indices for tumor recurrences vs. radiation-induced lesions

Fifty lesions were evaluated by ^18^F-FET PET analysis (Table 1). Median TBR_max_ was 2.9 in tumor recurrences (range 1.5–4.3) and 2.0 in radiation-induced lesions (range 1.2–3.5; *p* < 0.001). The median TBR_mean_ was 2.2 in tumor recurrences (range 1.4–2.8) and 1.7 in radiation-induced lesions (range 1.0–2.4; *p* < 0.001).

### ROC analysis of SUV

Receiver-operating-characteristic (ROC) curve analyses revealed a TBR_max_ of 2.15 as optimal cut-off value, leading to a sensitivity of 86 % and specificity of 79 % (accuracy 82 %, AUC 0.84, CI 0.73–0.96, *p* < 0.001). For the TBR_mean_, optimal cutoff was 1.95, equally leading to a sensitivity of 86 % and specificity of 79 % (accuracy 82 %, AUC 0.85, CI 0.74–0.96, *p* < 0.001).

According to this cut-off value, ^18^F-FET PET was false negative in 6 % of cases and false positive in 12 % of cases. In the false negative cases (*n* = 3), a median TBR_max_ of 1.9 (range 1.5–2.1) and a TBR_mean_ of 1.7 (range, 1.4–1.9) were found, median time from radiotherapy to ^18^F-FET PET was 4.1 months (range, 2.4–5.3 months). In the false positive cases (*n* = 6), a median TBR_max_ of 3.1 (range 2.6–3.5) and a TBR_mean_ of 2.2 (range 2.0–2.4) were seen (median time from radiotherapy to ^18^F-FET PET 10.4 months (range 2.5–23.5 months)).

### Evaluation of time activity curve patterns and TTP_min_

**Fig. 1:**
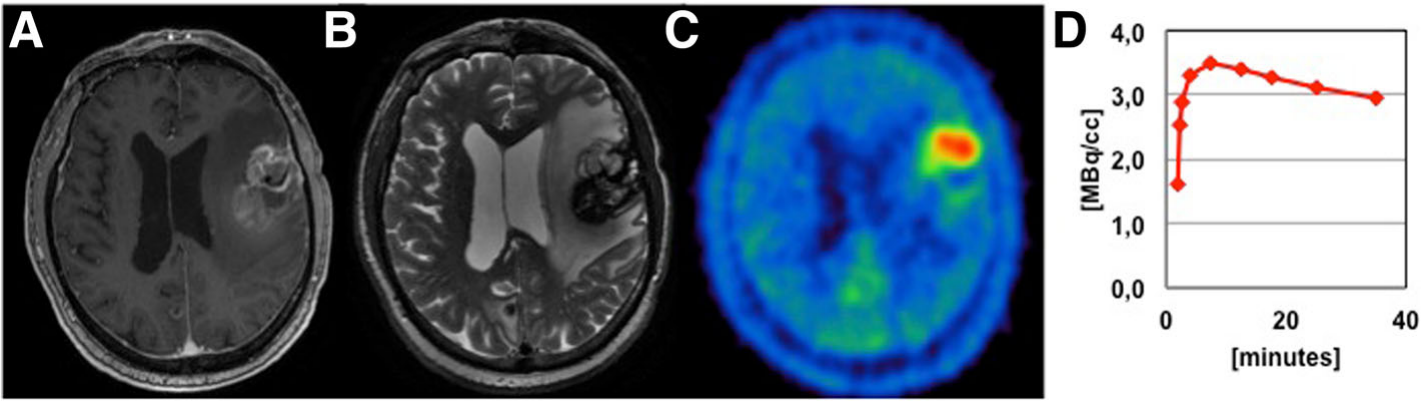
MRI and ^18^F-FET PET findings in a recurrent brain metastasis after focused high dose radiotherapy (Patient nr. 30). A ring-like contrast enhancement in the T1-weighted images (**a**), as well as an extensive edema in the T2-weighted images is seen (**b**). Both a focal ^18^F-FET uptake (TBR_max_ 3.5, TBR_mean_ 2.3) and decreasing TACs can be observed in static and dynamic PET analysis (**c**, **d**)

**Fig. 2:**
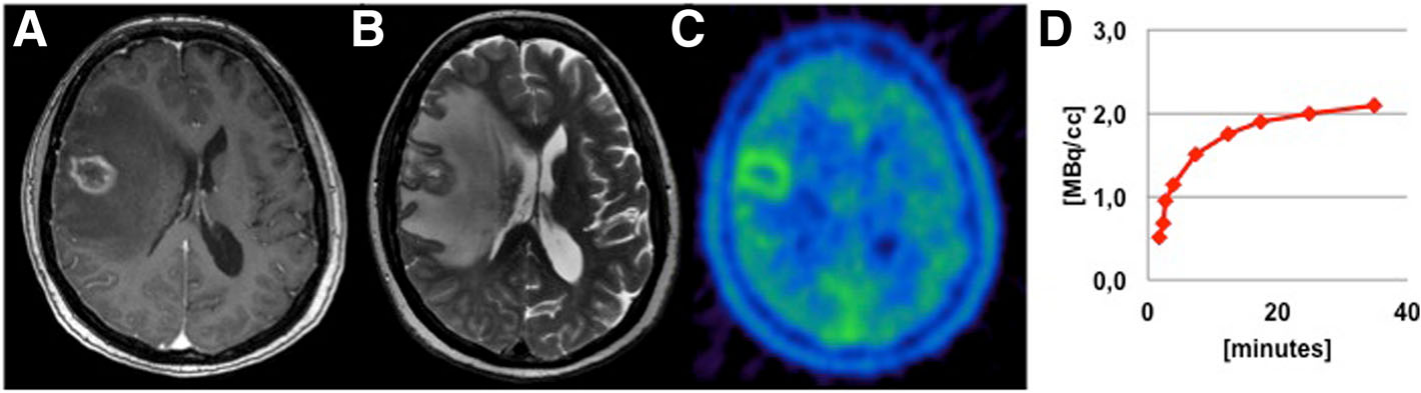
MRI and ^18^F-FET PET findings in a radiation-induced lesion after focused high dose radiotherapy (Patient nr. 18). A ring-like contrast enhancement in the T1-weighted images (**a**), as well as an extensive edema in the T2-weighted images is seen (**b**). Both a marginal focal ^18^F-FET uptake (TBR_max_ 1.4, TBR_mean_ 1.3) and increasing TACs can be observed in static and dynamic PET analysis (**c**, **d**)

Analysis of TAC patterns was performed in lesions with increased ^18^F-FET uptake (TBR_max_ ≥ 1.6) and was available in 42/50 cases. Increasing TACs were seen in 18 cases which were mainly associated with radiation-induced lesions (14/18 cases, 78 %), while decreasing TACs were found in 24 cases, of which 16 (67 %) were tumor recurrences (*p* = 0.01; two illustrative cases are given in Fig. 1 and 2). TTP_min_ was significantly shorter in tumor recurrences (median 17.5 min) than in radiation-induced lesions (median 35 min; *p* = 0.007). Although most of the cases with short TTP_min_ ≤ 12.5 min had a tumor recurrence (9/12 cases, 75 %) and most of cases with late TTP_min_ of 35 min were radiation-induced (12/15 cases, 80 %), no reliable cut-off TTP_min_ could be defined, as the rate of tumor recurrence and radiation-induced lesion in lesions with an intermediate TTP_min_ of 17.5–25 min was 53.3 % vs. 46.7 %. Sensitivity for the detection of tumor recurrence by the mere qualitative classification using decreasing TACs as indicator for tumor recurrence was 80 %, specificity was 63 % (accuracy 71 %, AUC 0.72, CI 0.55–0.88, *p* = 0.02). By adding dynamic to static PET information, the sensitivity and specificity for the detection of tumor recurrence could be increased: for lesions with TBR_max_ > 2.15 / TBR_mean_ > 1.95 in combination with decreasing TACs a sensitivity of 93 % and specificity of 83 % were obtained (accuracy 87 %, AUC 0.79, CI 0.66–0.92, *p* = 0.001).

Static and dynamic PET findings are summarized in Tables 1 and 2. Nearly identical results could be achieved for the subgroup of the 34 lesions undergoing a single ^18^F-FET PET evaluation (data not shown).

**Table 2:** Results of diagnostic performance

Identification of recurrent tumor	TBR_max_ > 2.15	TBR_mean_ > 1.95	Decreasing TACs	Decreasing TACs in combination with TBR_max_ > 2.15/TBR_mean_ > 1.95
Sensitivity	86 %	86 %	85 %	93 %
Specificity	79 %	79 %	60 %	84 %
Accuracy	82 %	82 %	74 %	88 %

## Discussion

Differentiation between radiation-induced lesions and tumor recurrence after focused high dose radiotherapy of brain metastases is challenging [8, 15]. In the current series, nearly 60 % of the metastases exhibiting clinical and/or radiological signs of tumor progression turned out to be radiation-induced lesions. In this context, the place of metabolic imaging and suitable radiotracers still needs to be defined. ^11^C-MET, ^18^F-DOPA and ^18^F-FET PET seem to be promising candidates for clinical routine [10, 11, 22, 23]. The use of ^11^C-MET however, is limited by logistic disadvantages (half life of 20 min restricting the use to sites with an on-site cyclotron [11]. Regarding ^18^F-DOPA, Lizzaraga and Cicone could recently show sensitivity and specifity values between 81.3 and 92.3 % rendering ^18^F-DOPA a promising radiotracer worthy of further examination [22, 23]. Using ^18^F-FET PET, Galldiks et al. have found sensitivity, specificity and accuracy values of 95, 91 and 93 %, respectively for tumor detection after radiotherapy of brain metastases in case of both TBR_mean_ values >1.95 and decreasing TACs [10]. Our results are in line with this study. This is especially true for the congruent TBR_mean_ cut-off value of >1.95, rendering this parameter clinically valuable due to its applicability in different sites. Interestingly, the TBR_mean_ threshold was identical despite differences in pretreatment between the two studies (exclusive focused high dose radiotherapy in our study versus mixed pretreatment with whole brain radiotherapy in the study by Galldiks). This might indicate that focused high dose radiotherapy itself determines the threshold values in PET imaging and that additional pretreatment with whole brain radiotherapy might not have a profound impact on TBR values.

Of note, compared to the above-mentioned study by Galldiks and colleagues, a lower cut-off value for TBR_max_ was found in our series (2.15 vs. 2.55). This might be explained by differing image reconstruction protocols, i.e. filtered-back projection in our study versus iterative reconstruction in the above-mentioned study. Lately, iterative reconstruction has been reported to be associated with higher TBR_max_ values in gliomas compared to filtered-back projection [24]. Therefore, the reported TBR_max_ cutoff values can possibly only be used with the same image reconstruction parameters, while the TBR_mean_ cut-off value seems to be more stable.

Our findings uncover diagnostic pitfalls after focused high dose radiotherapy of brain metastases. Our false positive cases (6/50) had markedly high TBR values (median TBR_max_ 3.1, range 2.6–3.5; median TBR_mean_ 2.2, range 2.0–2.4). Pretreatment was SRS in 4 cases and SBT in 2 cases and latency between ^18^F-FET PET and treatment ranged from 2.5–23.5 months (median 10.4 months). With regard to these parameters, the false positive cases did not differ from the correctly diagnosed tumor recurrences so that no explanation can be found for the apparently unspecific high ^18^F-FET uptake in these radiation-induced lesions. In our false negatives cases (3/50), the lesion size was remarkably small (median MR volume 0.6 ml, range 0.2–1.1 ml). In these cases, both the partial volume effect and the resolution have possibly contributed to the low TBR_max_ and TBR_mean_ values which were below the cutoff and therefore did not identify these small lesions as tumor tissue. In summary, these false positive/false negative result might anticipate a “lesional instability” leading to following clinical implications for the management of patients with brain metastases after focused high dose radiotherapy: i) suspicious lesions with high TBR values exceeding the cutoff should be histologically evaluated to verify tumor recurrence and to avoid overtreatment; ii) suspicious lesions with low TBR values below the cutoff do not need to be evaluated histologically and can be observed, however, iii) when being of small volume, should undergo closer follow-up as partial volume effects might hamper the PET evaluation. Furthermore, our observed rate of radiation-induced lesions as function of time implies that the risk for a radionecrosis is highest within the first two years after high-dose radiotherapy. Therefore, the time interval between radiotherapy and suspicion of tumor relapse should be taken into account in the further treatment rationale especially within the first two years.

Eventhough TTP_min_ was significantly shorter in tumor recurrences as compared to radiation-induced lesions, no reliable cutoff could be established for this parameter currently diminishing its prognostic impact. More data are necessary to determine its place within the diagnostic platform of brain metastases.

It should be stressed that not all patients had undergone stereotactic biopsy for histopathological verification (this was considered unethical in patients with suspected stable radiation-induced lesions). Systematic evaluation of ^18^F-FET PET uptake patterns in untreated and already irradiated tumors under consideration of the primary tumor will certainly help to overcome data heterogeneity and might be realized in the framework of future multicentre trials. The still increasing number of cancer patients will act as a pressure into this direction.

## Conclusions

^18^F-FET PET appears to be an attractive tool in the differentiation of tumor recurrences and radiation-induced lesions and its place is clearly beyond structural imaging. The combination of static (TBRs) and dynamic (TACs) parameters shows high sensitivity, specificity and accuracy values. Still, ^18^F-FET PET should be considered an addition rather than a replacement of stereotactic biopsy and close follow-up. This is especially true for selected ambiguous cases to avoid overtreatment or undertreatment.

## Abbreviations

^11^C-MET: L-Methyl-^11^C-Methionine
^18^F-FDG: ^18^F-2-Deoxy-2-Fluoro-D-Glucose
^18^F-FET PET: O-(2-^18^F-Fluoroethyl)-L-Tyrosine
BBB: Blood brain barrier
CT: Computerized tomography
FLAIR: Fluid attenuated inversion recovery
MRI: Magnetic resonance imaging
ROC: Receiver operating characteristic
SBT: Stereotactic iodine-125 brachytherapy
SRS: Stereotactic radiosurgery
SUV: Standardized uptake value
TAC: Time activity curve
TBR_max_: Maximum tumor-to-background ratio
TBR_mean_: Mean tumor-to-background ratio
TTP_min_: Minimal time to peak
